# Effect of botulinum toxin injection on length and force of the rectus femoris and triceps surae muscles during locomotion in patients with chronic hemiparesis (FOLOTOX)

**DOI:** 10.1186/s12883-018-1110-8

**Published:** 2018-08-02

**Authors:** Anthony Supiot, Maxime Geiger, Djamel Bensmail, Phillippe Aegerter, Didier Pradon, Nicolas Roche

**Affiliations:** 1 Inserm Unit 1179, Team 3: Technologies and Innovative Therapies Applied to Neuromuscular diseases, UVSQ, CIC 805, Physiology–Functional Testing Ward, AP-HP, Raymond Poincaré Teaching Hospital, Garches, France; 2 Assistance Publique-Hôpitaux de Paris, Hôpital Ambroise Paré, Unité de Recherche Clinique (URC), Boulogne, France; 3 CIAMS, University Paris-Sud, Université Paris-Saclay, 91405 Orsay Cedex, France; 4 CIAMS, Université d’Orléans, 45067 Orléans, France

**Keywords:** Stroke, Botulinum toxin type A, Muscle length, Motion analysis, Gait

## Abstract

**Background:**

After stroke, spasticity of the rectus femoris (RF) and triceps surae (TS) muscles frequently alters the gait pattern. Knee flexion and ankle dorsiflexion in swing are often reduced, respectively called Stiff Knee Gait (SKG) and equinus. A preliminary uncontrolled study suggested that botulinum toxin type A (BTX-A) injections could improve muscle length and force generated during gait, improving inter-segmental coordination.

The aim of this randomised controlled study is thus to evaluate changes in the length of the RF and TS muscles during gait 1 month after either BTX-A or placebo injection in patients with chronic stroke, SKG and spastic equinus. The secondary aims are to evaluate peak length and peak force generated during gait, as well inter-segmental coordination assessed using the continuous relative phase method initially described by Barela et al. in patients with stroke.

**Methods:**

This is a prospective, three-centre, randomised, placebo-controlled, triple blind study over 3 months with 4 visits. Forty patients will be included. During visits V1, V3 and V4, length and force generated by RF and TS during gait will be assessed using musculoskeletal models (MSM). Muscle force will also be assessed using an isokinetic dynamometer. Inter segmental coordination will be evaluated using 3D gait analysis and functional tests will be performed. During V2, patients will receive either an injection of BTX-A in the RF and TS muscles or a placebo injection of saline solution.

**Discussion:**

We expect an increase in peak length and a decrease in peak force generated by the RF and TS muscles in the BTX-A group 1 month post injection. Moreover, we expect these parameters to be more improved in the BTX-A than the Control group. This is the first study to assess these parameters in a randomised, controlled trial using instrumented methods (isokinetic evaluation and 3D gait analysis). The results should help to improve understanding of the mechanism(s) underlying improvements in inter-segmental coordination that have been found in many previous uncontrolled studies.

**Trial registration:**

ClinicalTrials.gov: NCT01821573, First received: March 27, 2013 Last updated: September 14, 2016 Last verified: September 2016 Other Study ID Numbers: P110136 AOM11223.

## Background

Around 85% of stroke survivors regain the capacity to walk. However the kinetic and kinematic parameters of post-stroke gait are typically altered, with a reduction in cadence, stride length and velocity [1–3]. Stiff knee gait (SKG), characterized by decreased knee flexion during swing phase [4], and equinus (reduced ankle dorsiflexion) [5] are common post-stroke gait patterns.

The loss of knee flexion during swing can be attributed to several mechanisms, including weakness of the hip flexor muscles, a reduced plantar flexion moment and inappropriate activity of part of the quadriceps muscle, particularly the Rectus Femoris (RF) [4]. The bi-articular RF is normally active during the final phase of double support (end of stance, beginning of swing) to control knee flexion. If it is inappropriately activated during swing, it restricts knee flexion during this phase [4, 6, 7].

The loss of dorsiflexion can be secondary either to weakness of the dorsiflexor muscles, particularly the Tibialis anterior (TA), or to inappropriate activity of the Triceps Surae (TS) muscles [8, 9]. Inappropriate activity of the TS reduces dorsiflexion and results in initial contact with the foot in plantar flexion.

Medical interventions to improve gait mostly target the inappropriate activity of the RF and TS. Botulinum toxin type A (BTX-A) is a common treatment to reduce muscle activity. It blocks the liberation of neurotransmitters at the presynaptic neuromuscular junction, inducing focal, transitory and reversible paralysis of the injected muscle. The effect lasts around 3 months [10].

Several uncontrolled studies have evaluated the effects of intramuscular BTX-A injection on gait, showing increases in knee flexion of 5 to 8 degrees following the treatment [6, 11, 12]. Knee flexion velocity [11] and gait velocity [6] have also been found to increase with BTX-A, along with a reduction of impairment (Stroke Impairment Assessment Set) and an improvement in gait capacity (Abiloco scale) [12]. BTX-A injection has been shown to improve coordination between the hemiparetic thigh and shank [13], associated with a reduction of compensatory behaviour in the non-hemiparetic limb [14]. In addition, a pilot study using a musculoskeletal model (SIMM ®) in 10 patients with stroke [15] showed that peak normalised RF length increased following BTX-A injection, as did peak and mean lengthening velocity. The SIMM ® musculoskeletal model can also be used to assess peak muscle force developed during gait, however this has never been done in patients with stroke.

The effectiveness of BTX-A injections in the TS has also been demonstrated. A randomised placebo-controlled study of 234 patients with stroke showed that BTX-A injection in the TS reduces passive resistance to ankle dorsiflexion (Modified Ashworth), pain, and dependence on assistive devices (orthoses and walking sticks) [5]. More recent studies have shown that BTX-A injection of the TS also increases gait speed [16, 17]. BTX-A injection combined with non-pharmacological treatments such as functional electrical stimulation, stretching and taping, increases ankle dorsiflexion in stance in adults with spastic equinus [18].

There is therefore much evidence to show that BTX-A injection in the RF or TS muscles reduces spasticity and improves gait kinematics, however the effect on length of the RF muscle has only been evaluated in one pilot study [15].

Based on the results of the pilot study by Lampire et al. [15], we hypothesise that BTX-A injection into the spastic RF and TS muscles of patients with chronic hemiparesis i) will improve peak length and mean lengthening velocity of the RF and TS and ii) will increase peak length and lengthening velocity of these muscles during gait.

Muscle force generated during gait has never been evaluated using a musculoskeletal model after BTX-A injection. Because BTX-A injection blocks transmission at the neuromuscular junction and is known to reduce muscle force evaluated by static tests, we believe it is important to evaluate the effect of BTX-A on force generated during gait. We hypothesise that force produced by the RF and TS muscles during gait will be decreased following BTX-A injection but not after the placebo injection.

The main aim of this randomised, placebo-controlled, triple blind study is to evaluate the effect of BTX-A injection in the RF and TS muscles on their peak length during gait in patients with chronic stroke.

The secondary aims are to: i) evaluate peak force generated by the RF and TS during gait following BTX-A injection, ii) evaluate changes in inter-segmental coordination during gait following BTX-A injection, iii) evaluate the impact of BTX-A injection on quality of life and iv) compare the effect of BTX-A injection with a placebo injection on the parameters specified above, in patients with chronic stroke.

The third aims are to: i) evaluate the quality of life, ii) evaluate the functional independence in motor and cognitive domains, iii) evaluate the locomotor ability.

## Method/design

### Ethical approval

All subjects included will provide written informed consent for participation. This study will be performed in accordance with the ethical codes of the World Medical Association and has been approved by the local ethics committee (reference number: AOM 11223-P110136 6 FOLOTOX/ 2012–003168-34 ref. CPP 12064 ClinicalTrials.gov: NCT01821573).

### Study design

The study will be prospective, 3-centre, randomised, placebo-controlled and triple blinded. The total duration of the study will be 36 months. The duration of participation of each patient will be 3 months, with four visits. The patients and the injecting doctors and assistants, as well as the evaluators will be blinded to the patients’ group allocations. Randomization will be carried out by computer software. During visits V1, V3 and V4 the measurements will be carried out throughout the day with breaks allowed between each (Table 1).

**Table 1:** Tests and evaluations during each visit

Tests and evaluations	V1	V2	V3	V4
Inclusion/ exclusion criteria	X			
Informed consent	X			
Randomisation	X			
Injection BTX-A or placebo		X		
Evaluation of passive range of motion (MG)	X		X	X
Evaluation of spasticity (MAS)	X		X	X
Evaluation of strength (MRS)	X		X	X
SF 36	X		X	X
FIM	X		X	X
ABILOCO scale	X		X	X
Berg balance scale	X		X	X
Abc scale	X		X	X
Timed up and go	X		X	X
3D analysis	X		X	X
Isokinetic evaluation	X		X	X

*V1* visit 1, *V2* visit 2, *V3* visit 3, *V4* visit 4, *MA* manual goniometer, *MAS* modified Ashworth, *MRC* Medical Research Council, *SF36* The Short Form (36) Health Survey, *FIM* Functional independence measure, *3D analysis* gait motion analysis. All outcome measures will be evaluated during the first visit (baseline-V1) and 1 month (V3) and 3 (V4) months after injection. All evaluations will be performed in the same order at each session in order to limit bias related to fatigue

### Participants

Patients will be recruited from routine medical consultations in the participating hospitals. For this study, stroke is defined according to the World Health Organisation as a rapid onset event of vascular origin reflecting a focal disturbance of cerebral function, excluding isolated impairments of higher function and persisting longer than 24 h [19].

### Inclusion criteria

Adults over the age of 18 years with unilateral chronic stroke that occurred more than 6 months previously and who are able to walk 10 m without any assistive devices will be included. They must have a loss of peak knee flexion during the swing phase of the gait cycle due to spasticity of the RF muscle. The ankle must also remain in plantarflexion during swing due to spasticity of the TS. The severity of RF and TS spasticity of the affected limb will be determined using the Modified Ashworth Scale (MAS). Subjects will be included if they have a spasticity equal or upper of 1. Patients must give informed consent and females must be taking oral contraception.

### Exclusion criteria

Participants will not be included if they have aphasia, have undergone lower limb surgery less than 6 months previously, for example muscle-tendon lengthening, ankle foot arthroplasty, RF tenotomy with or without transfer, or any surgery aimed to improve gait, if they have other diseases or pre-existing neuro-muscular disorders, if they are pregnant, if they had an injection of BTX-A less than 3 months previously, if they had a previous hypersensitive reaction to BTX-A or are taking aminoglycosides.

### Randomization method

All patients included are routinely followed in the spasticity-units of the three participating centers. All will already be treated with BTX-A and will have undergone regular spasticity ratings using the MAS. Patients with spasticity of both the RF and TS will therefore be identified before the study is presented to them during visit 1. In addition, all patients will already have undergone routine 3D gait analysis as part of their management, thus the decrease in peak knee flexion and peak ankle plantarflexion in swing phase will already be documented, with the cause reported as RF and TS spasticity. Randomization will be carried out using randoWeb software (http://randoweb.aphp.fr) at the beginning of visit 2, then either BTX-A or the placebo will be injected according to group allocation. To avoid problems relating to expiration of the BTX-A, the BTX-A and placebo packs will be randomized, not the patients. A randomization list will be uploaded on a dedicated server (CleanWeb) and each patient will be attributed an injection pack number. The randomization will be block-balanced. Patients will thus be injected with either BTX-A or the placebo at visit 2.

### Intervention group

Previous studies [6, 12, 15, 20] demonstrated that 200 U of BTX-A injected into the RF could improve RF length, spatio temporal gait parameters and intersegmental coordination. Pradon et al. showed that BTX-A injected in the TS (total of200 U: 75 U in each gastrocnemius and 50 U in the soleus) combined with use of an ankle foot orthosis improved gait parameters [20]. Caty et al. demonstrated that 200 U of Botulinum toxin type A (Botox) in the TS improved stiff knee gait and gait capacity [12].

The doses determined for the present study have thus been based on those studies as well as French Health Authority guidelines. Patients in the BTX-A group will receive a total of 350 units of BTX-A (Botox®, Allergan Inc). BTX-A is in powder form (BOTOX® 100 units Allergan). The BTX-A powder will dissolve with NaCl (0.9%) solution to have four syringes 1) 150 units (3 ml) for an injection in the RF in 3 points, 2) 70 units (1.4 ml) for the medial gastrocnemius in 1 point, 3) 70 units (1.4 ml) for the lateral gastrocnemius in 1 point, and 4) 60 units (1.2 ml) for the soleus in 3 points***.***For injection into the RF, the first point will be 5 cm below the inguinal crease, on a line between the antero-inferior iliac spine and the patella. The second point will be ten centimetres above the patella and the third on the same line, half way between the first two points [13, 15]. For injection into the triceps surea, the French Health Authority guidelines will be followed [21]. For the gastrocnemii, the injection points are on a horizontal line separating the upper 1/4 from the lower 3/4 of the lower leg. For the soleus, the proximal injection points are on a horizontal line separating the upper 2/5 and lower 3/5 of the lower leg and the distal point is on a line separating the upper 2/3 from the lower 1/3 of the lower leg.

### Placebo group

For placebo group a solution of NaCl (0.9%) will use as a placebo. In addition the patients will receive identical volumes of NaCl (0.9%) that intervention group. Identically the placebo will be conditioned in four syringes: 1) 3 ml for the RF in 3 points, 2) 1.4 ml for the medial gastrocnemius in 1 point, 3) 1.4 ml for the lateral gastrocnemius in 1 point, and 4) 60 units (1.2 ml) for the soleus in 3 points. The injection points will be identical to those used for the intervention group.

All injections (both groups) will be carried out under electrostimulation guidance with a stimulation intensity of 5 mA. Visit 2, during which BTX-A or placebo injection will be performed, will be either on the same day as Visit 1 after randomisation, or within 7 days.

### Blinding

In order to ensure study blinding, only the pharmacist of each centre will know the nature of the box (BTX-A or placebo). Moreover, an independent nurse will prepare the syringe in a separate room.

### Clinical evaluation

All patients will undergo a comprehensive clinical evaluation. To limit inter-investigator variability, one physiotherapist in each centre will carry out all assessments. In addition, this physiotherapist will be blinded to group allocation.The clinical examination will include passive range of motion (ROM) of the hip, knee and ankle using a manual goniometer. Measurement of joint ROM will be carried out according to the protocol recommended by Clarkson [22]. Spasticity will be evaluated using the Modified Ashworth Scale (MAS) [23]. This scale has been validated for the evaluation of spasticity [24, 25] and is sensitive to change following BTX-A treatment [26]. Lastly, strength of the hip extensor, flexor and abductor, knee flexor and extensor, ankle dorsiflexor and plantarflexor muscles of the affected and non-affected lower limbs will be measured using the Medical Research Council (MRC) scale [27, 28] (Table 2).

**Table 2:** Summary of outcome measures for each study aim

Aims	Parameter evaluated	Outcome measure
First	Peak length of RF and TS during gait	3D analysis
		MSM
	Peak strength of RF and TS	Isometric evaluation
Second	Inter-segmental gait coordination	3D analysis:
		EMG analysis
		Muscle synergies
		Peak joint angles
		Peak joint torques
		Inter joint coordination
		ABILOCO scale
		TUG
		6MWT
		10MWT
		Stairs test
	Balance	Berg balance scale
		ABC scale
Third	Independence	FIM
	Quality of life	SF 36

*RF* rectus femoris, *TS* triceps surae, *TUG* Timed up and go test, *6MWT* Six minute walk test, *10 MWT* Ten meter walk test, *ABC scale* Activities Balance Confidence scale, *FIM* Functional independence measure, *SF 36* Short form (36) health survey, *3D analysis* gait motion analysis, *MSM* Musculoskeletal models

## Outcome measures

### Primary aim: Outcome

The primary outcome is change in maximal length of the injected muscles during gait one month after injection of BTX-A or placebo, quantified by 3D motion analysis and a musculo-skeletal model. It will be determined by comparison of the results of the evaluations carried out at V1, V3 and V4.

#### Musculoskeletal model

Musculoskeletal models (MSM) have been used in many studies since the 1980’s [29–34] to simulate the mechanical properties of muscle contraction. This procedure has been validated, particularly for the TS muscle [35]. Kinematic, kinetic and EMG data recorded during 3D gait analysis are inputted into the model for the calculation of biomechanical muscle parameters during movement. MSM thus provide information regarding muscle function during normal and pathological gait [36–40]. SIMM® (Software for Interactive Musculoskeletal Modeling) software will be used to calculate the three biomechanical muscle properties of interest in the present study. The standard generic SIMM® model [30] uses data from the gait analysis. The model is constituted of thirteen rigid segments with seventeen degrees of freedom and each limb is made up of forty-three muscle-tendon complexes. Each complex is defined by its origin, insertion, and if necessary, by via-points in order to specify the trajectory of the muscle. SIMM® uses static optimization based on inverse dynamics, with a constraint based on the EMG envelope to calculate muscle forces.

#### Calculation of length

Normalized peak length will be defined as: peak muscle length during gait minus muscle length in the ‘virtual’ anatomical reference position, in millimetres [15]. This normalization reduces bias related to morphological differences between subjects, as well as differences related to marker placement between different visits for each patient.

Peak muscle lengthening velocity will be calculated for each gait cycle and mean peak lengthening velocity will be then calculated for each subject.

### Secondary aim: Outcomes

The secondary outcomes are peak muscle force generated during gait assessed by the MSM, peak force measured during the isometric evaluation, inter-segmental coordination during gait assessed by 3D analysis, and functional tests.

#### Peak force during gait

Peak force developed during gait will be calculated and expressed as a percentage of body weight in order to take into account differences in the magnitude of muscle forces between subjects.

The instant of the gait cycle (as a % of the gait cycle, normalised according to the toe off) at which the peak force of each muscle occurs will be determined. A negative value indicates that the peak force occurred in stance phase, while a positive value indicates that it occurred in swing phase.

#### Maximal strength

A CON-TREX isokinetic dynamometer will be used to evaluate force. The physiotherapist carrying out this evaluation will be blinded to group allocation. A standardized sitting position on the dynamometer will be used with the trunk fixed by a belt to the back rest to ensure that the position is maintained throughout the test. Participants will be asked to place their hands on their thighs and to keep the upper limbs relaxed throughout the test. To avoid any mal-alignment of axes, limit compensatory body movements and eliminate the degrees of freedom of the other joints, the non-paretic lower leg will be blocked and the thigh of the tested leg will be fixed. Care will be taken to align the axes of rotation of the joints of the tested limb with the axes of rotation of the dynamometer. All settings will be noted in order to ensure the position is identical across sessions for each subject.

The peak force couple produced by the knee flexors and extensors during active movements of knee flexion and extension will be calculated, as well as the angle at which the peak force couple occurs during passive movements. An isometric evaluation of maximum force developed by the quadriceps and hamstring muscles will also be carried out with the knee flexed to 90 degrees [41].

#### Inter-segmental coordination during locomotion

Inter segmental coordination during gait will be analyzed using a 3-dimensional (3D) motion capture system (Motion Analysis Corporation, Santa Rosa, CA, USA, six cameras, Sampling Frequency 100 Hz) and two force plates (AMTI, Watertown, MA, USA, Sampling Frequency 1000 Hz). The trajectories of 24 markers placed on anatomical landmarks using the Helen Hayes marker set will be recorded and filtered using a fourth-order zero-lag Butter worth low-pass-filter with a 6 Hz cut off frequency.

3D Gait Analysis will be carried out during V1, V3 and V4. To limit inter-investigator variability, one operator in each centre will carry out all assessments. In addition, this operator will be in blinded to group allocation. Two conditions will be analysed: preferred and maximal walking speed. For each condition, eight trials will be recorded to ensure a minimum of ten gait cycles from both lower limbs. Boudarham et al. suggested that during 3D analysis, there is a phase of adaptation during which gait is unstable [42]. They thus suggested kinematic and kinetic analysis should be based on three to nine gait trials in patients with hemiparesis.

OrthoTrack 6.5 software (Motion Analysis Corporation, Santa Rosa, CA, USA) will be used to calculate kinematic and kinetic parameters, using Euler rotations (Grood and Suntay method) for the kinematic parameters.

Spatio-temporal parameters will be calculated for both lower limbs, including gait velocity, cadence, step and stride length, step width, and the duration of the single support phase. Kinematic and kinetic parameters will be calculated for both lower limbs for each sub-phase of the gait cycle (initial double contact phase, single support phase, final double contact phase and swing phase). The main kinematic parameters are peak flexion, extension, peak abduction, adduction, valgus/varus and rotation as appropriate for the pelvis, hips, knees and ankles. Ground reaction force and peak joint moments of the hips, knees and ankles will be calculated in the three dimensions of space.

The activity of eight muscles will also be recorded using a surface EMG system (MA311, Motion Lab Systems, Baton rouge; band-pass 15-3000 Hz): rectus femoris (RF), vastus lateralis (VL), gluteus maximus (GMax), hamstring (HAM), biceps femoris (BF), soleus (SOL), medial gastrocnemius (MG), tibialis anterior (TA). Electrode placements will follow SENIAM recommendations [43]. These muscles function in synergies to produce gait, therefore an analysis of muscle synergies will be performed. Evaluation of the coordination of lower limb segments during gait typically involves reducing all the degrees of freedom to a small number of patterns [44] that reflect the underlying neural control of gait [45]. To extract muscle synergies, we will use the model presented in Delis [46]. We will use the associated sNM3F algorithm to obtain a space-by-time modular description of EMG patterns. According to that method, modularity is defined as “linear combinations of both spatial and temporal modules”. This model has been shown to unify and encompass most previous approaches and to yield a concise description of muscle patterns underlying the execution of various motor tasks. Muscle synergies will be extracted from the EMG recorded during the gait analysis (RF, VL, HAM, GAS and SOL) using spatial and temporal methods in order to evaluate changes in the pattern of muscle activity following BTX-A or placebo injection and the subsequent reduction (or not) of spasticity and force produced. The relationship between muscle force and type of synergy developed by patients will be then be evaluated.

Five functional tests will be used to evaluate the impact of any changes in intersegmental coordination on functional ability. These tests will be carried out by the blinded physiotherapist.

The Timed up and go test (TUG): This test consists of measuring the timed performance of rising from a chair, walking 3 m, turning and sitting again. The subject wears his/her usual shoes and may use his/her usual gait aid. The starting position is sitting with the back against the backrest of the chair, the arms resting on the chair arms and the gait aid within easy reach. Time begins when the subject’s back is no longer in contact with the chair back and stops when the subject is sitting once again with his/her back against the chair back.

The 6 min walk test (6MWT): Subjects will be asked to walk for 6 min and the distance covered will be measured. They may slow down or stop but must resume walking as soon as possible. A standardized sentence of encouragement will be used to inform the patient of the remaining time each minute. This test will be carried out in a 30 m long, unused corridor. A cone will be placed at each end around which the subject must turn [47, 48].

Ten meter walk test (10 MWT): The time taken to walk 10 m at maximal velocity will be recorded. The patients will be asked to walk as fast as possible over a distance of 14 m. The first and last 2 m will not be counted in order to eliminate the phases of acceleration and deceleration. Subjects will wear their usual shoes and may use a gait aid. Three trials will be carried out and the average used for analysis [49, 50].

Stairs test (ST): Subjects will be asked to ascend and descend a flight of 13 stairs (15 cm high) 3 times at maximal speed, and the mean will be used for analysis. The starting point will be a line 16 cm away from the first step. After each ascent or descent, the subject will be allowed to rest. Subjects can use the handrail but only for balance, and must not pull on it. The time will begin when the first foot leaves the ground and will stop when the last foot touches the ground [51].

The berg balance scale (BBS) will be used to measure balance [52].The BBS has been developed to measure static and dynamic equilibrium in adults to detect people at risk of fall. It can also be used to identify people who can walk unaided and to predict the difficulties some individuals may encounter in everyday activities. The BBS includes 14 tests that assess static balance and dynamic equilibrium as: unipodal support trunk rotation getting up and sitting.

### Third aim: Functional outcomes

Quality of life will be evaluated using the short form-36 (SF 36) [53, 54].The Functional Independence Measure (FIM) [55] will be used to evaluate functional independence in eighteen motor and cognitive domains. The FIM can be used to follow change from the beginning of rehabilitation to discharge [56]. The ABILOCO scale (a measure of locomotor ability for adults) [57] will be used to be assess locomotor ability. The test rates the individual’s ability to move about effectively in his/her environment by the evaluation of thirteen tasks including simple and complex movements. The scale is sensitive to changes in walking ability [12].The ABC scale (Activities Balance Confidence) will be used assess confidence in carrying out sixteen activities of daily living both indoors and outdoors [58].

### Management of adverse events

Patients will be informed about possible adverse events at the time of inclusion. If any adverse events occur, the study coordinator must be immediately contacted. Two types of adverse events will be considered.

Non-serious adverse events or effects are defined as any harmful manifestation that occurs in the subject, whether or not this manifestation is linked to any experimental element of the research or products used. Non-serious events may include: pain or burning at the injection site, injected limb pain, ecchymosis, hypertonia, muscular weakness, arthralgia, asthenia, hyperesthesia, pain, depression, insomnia, discomfort, nausea etc.

Serious adverse events or effects are any adverse event or effect that results in death, endangers life, requires hospitalization or prolongation of hospitalization, causes significant or lasting disability, or results in an anomaly or a congenital malformation. Undesirable effects whose nature, severity or evolution does not correspond to the information contained in the references recognized by the authorities are considered as serious, as are any adverse effects related to the diffusion of the BTX-A away from the injection site. Serious adverse events therefore include excessive muscle weakness, dysphagia, inhalation pneumonia, anaphylactic reactions etc.

## Statistical analysis

### Sample size calculation

Sample size was estimated using G*power software [59]. The input data were taken from Lampire et al. [15] who assessed the effect of BTX-A injection on length and lengthening velocity of rectus femoris during gait in chronic stroke patients). Using a two-tailed test at a threshold of 5% and a power of 80%, a sample of seventeen subjects per group was found to be necessary [60]. We therefore plan to recruit forty patients to take into account potential patients lost to follow-up. The study is currently underway. On the first of April 2018, twenty-eight participants had been included.

### Statistical analysis

Data will be analysed using SAS 9.3 (Cary Inc., NorthCarolina, USA) and “R” software (v2.14 R Foundation for Statistical Computing, Vienna,Austria. http://www.R-project.org/).

#### Description

Patients will be described by their status: eligible, included, not included (with reason for non-inclusion), left the study, and completed evaluations, within each group and for each centre (CONSORT criteria). The data will be described according to group allocation (BTX-A or placebo) and time (visit), using the usual statistics for each type of variable.

A Chi-squared test will be used to compare the distributions of qualitative variables, and analysis of variance will be used to compare the distributions of continuous quantitative variables (the variables may be adjusted to improve the symmetry or stabilise the variance). Ordinal variables (or those that do not have a normal distribution) will be compared using the non-parametric Kruskal-Wallis test.

#### Analysis of primary outcome

Change in peak length of the RF and TS muscles during gait at 1 month (difference in length between V3 and V1 will be compared between groups using a Student t-test. To prevent the risk of false positives induced by multiple testing, a Bonferroni Holm correction will be applied to the *p*-values for peak length, peak force and peak strength [61]. The level of significance will be set at 0.05/3.

#### Analysis of secondary outcomes

The statistical test used for the primary outcome will also be used to evaluate the effect of BTX-A at 3 months (V4). To compare changes in the secondary criteria across visits between the two groups (change in peak length of injected muscles, joint angles, modified Ashworth score, functional scores and quality of life), repeated measures models will be used. Depending on the distribution of continuous or binary variables, mixed models or generalized estimation equations (GEE) will be used respectively. The models will include the treatment variable (BTX-A or placebo), time and interaction between time and treatment as explanatory factors. If a significant interaction is found, comparisons between both groups (BTX-A and placebo) will be carried out for each time-point using a Student t-test. In order to take in to account any effects of multiple testing, these outcomes will be considered as explanatory factors rather than determinants of the effectiveness of BTX-A.

#### Analysis of safety criteria

The number and percentage of patients who experience adverse expected or unexpected effects, as well as serious adverse effects relating to the active treatment will be analysed. The nature of each effect will be specified. The duration and percentages of any adverse effects following BTX-A injection will be compared with any that occur following placebo injection using non-parametric tests.

#### Sample analysed

Because this is a study of effectiveness, the main analysis will be carried out following intention to treat principles. The safety analysis will be carried out on all participants who received at least one injection, whatever the substance injected. For the safety analysis, participants will not be analysed according to treatment allocation, but according to the treatment actually received.

#### Missing, unused or invalid data

The status of the patients (included, randomised, evaluated as intention to treat, lost to follow-up, left the study or deviated from the protocol) will be described and compared for each treatment arm and centre. Patients lost to follow-up, deceased (which should be exceptional) or left the study will be censored at the date of the last information. In case of censure, the last known value of the result will be used. All missing or invalid data will be replaced by the last available data for the corresponding parameters for the patient. The “last observation carried forwards procedure” will be used. In case of a large quantity of missing data, sensitivity analysis using conditional imputation will be carried out.

#### Management of changes to the planned analysis

Changes or supplementary analyses could be carried out. They will be considered as “post-hoc” and will be reported as such in the analysis and publication. Any retrospective changes to the planned analysis will be validated by the Study Committee and justified in an amendment to the protocol and in the trial report.

The statistical analysis will determine if BTX-A: i) increases peak muscle length during gait; ii) reduces peak force generated; iii) improves inter-segmental coordination during gait and iv) improves performance on functional tests.The second aim is to determine whether the effects of BTX-A are maintained at V4 compared with V1, suggesting a remnant action of BTX-A.

## Discussion

We expect that BTX-A injection in the RF and TS muscles will increase their length and peak lengthening velocity and decrease peak muscle force during gait 1 month, and perhaps also 3 months, after the injection. We expect to find a reduction in the spasticity of these muscles, an increase in passive knee flexion and passive ankle dorsiflexion, a decrease in knee extensor and plantarflexor muscle strength (MRC scale, and isokinetic dynamometer) and a reduction in force generated by these muscles during gait (SIMM model) in the BTX-A group. The peak force couple produced by the knee flexors/extensors during active movements (measured on the isokinetic dynamometer) should decrease, and the angle at which peak force occurs during passive movements should increase, demonstrating a decrease in spasticity. We also expect spatiotemporal gait parameters to improve, with an increase in gait velocity, cadence and stride length. Peak ankle dorsiflexion in stance and perhaps also in swing, and peak knee flexion in swing should increase. Peak plantar flexion moment in pre-swing phase may decrease. Based on the results of the pilot study by Lampire [15], we hypothesise that the length and lengthening velocity of the injected muscles will improve 1 month post injection.

We anticipate that these changes (particularly 1 month after BTX-A injection) will be associated with a concomitant increase in gait velocity during the 10-MWT and distance walked during the 6MWT, and reductions in performance time of the stairs and Timed Up and Go tests.

This is the first randomised, placebo-controlled trial using instrumented measures, such as 3D gait analysis and an isokinetic dynamometer, to evaluate the effects of BTX-A on gait parameters and force in patients with chronic stroke-related hemiparesis. We expect improvements of all the parameters studied in the BTX-A group. In contrast, we do not expect any significant improvement of any outcome measures in the placebo group.

Analysis of correlations between the different results will reveal if changes in the biomechanical properties of the injected muscles are related to improvements in kinematic and kinetic parameters and functional tests.

The results of this study will therefore increase understanding of the results of many previous un-controlled studies that have found improvements in kinematic parameters following BTX-A injection in patients with chronic hemiparesis.

## Abbreviations

10MWT: Ten meter walk test
6MWT: Six minute walk test
ABC scale: Activities balance confidence scale
ABILOCO: A measure of locomotion ability for adults
BBS scale: Berg balance scale
BTX A: Botulium toxin type A
EMG: Electromyography
GEE: Generalized estimation equation
MAS: Modified ashworth scale
MRC: Medical research council
MSM: Musculoskeletal models
RF: Rectus femoris
SKG: Stiff knee gait
ST: Stairs test
TA: Tibialis anterior
TS: Triceps surae
TUG: Timed up and go test

## References

[1] Brandstater ME, Gowland C, Clark BM (1983). Hemiplegic gait: analysis of temporal variables. Arch Phys Med Rehabil.

[2] Olney SJ, Griffin MP, McBride ID (1994). Temporal, kinematic, and kinetic variables related to gait speed in subjects with hemiplegia: a regression approach. Phys Ther.

[3] Pinzur MS, Sherman R, Dimonte-Levine P, Trimble J (1987). Gait changes in adult onset hemiplegia. Am J Phys Med Rehabil..

[4] Kerrigan DC, Gronley J, Perry J (1991). Stiff-legged gait in spastic paresis. A study of quadriceps and hamstrings muscle activity. Am J Phys Med Rehabil.

[5] Pittock SJ, Moore AP, Hardiman O, Ehler E, Kovac M, Bojakowski J (2003). A Double-Blind Randomised Placebo-Controlled Evaluation of Three Doses of Botulinum Toxin Type A (Dysport&lt;sup&gt;®&lt;/sup&gt;) in the Treatment of Spastic Equinovarus Deformity after Stroke. Cerebrovasc Dis.

[6] Robertson JVG, Pradon D, Bensmail D, Fermanian C, Bussel B, Roche N (2009). Relevance of botulinum toxin injection and nerve block of rectus femoris to kinematic and functional parameters of stiff knee gait in hemiplegic adults. Gait Posture..

[7] Kerrigan DC, Roth RS, Riley PO (1998). The modelling of adult spastic paretic stiff-legged gait swing period based on actual kinematic data. Gait Posture..

[8] Kim CM, Eng JJ (2004). Magnitude and pattern of 3D kinematic and kinetic gait profiles in persons with stroke: relationship to walking speed. Gait Posture.

[9] Knutsson E, Richards C (1979). Different types of disturbed motor control in gait of hemiparetic patients. Brain J Neurol.

[10] Schantz EJ, Johnson EA (1992). Properties and use of botulinum toxin and other microbial neurotoxins in medicine. Microbiol Rev.

[11] Stoquart GG, Detrembleur C, Palumbo S, Deltombe T, Lejeune TM (2008). Effect of Botulinum toxin injection in the rectus Femoris on stiff-knee gait in people with stroke: a prospective observational study. Arch Phys Med Rehabil.

[12] Caty GD, Detrembleur C, Bleyenheuft C, Deltombe T, Lejeune TM (2008). Effect of simultaneous Botulinum toxin injections into several muscles on impairment, activity, participation, and quality of life among stroke patients presenting with a stiff knee gait. Stroke.

[13] Hutin E, Pradon D, Barbier F, Gracies J-M, Bussel B, Roche N (2010). Lower limb coordination in Hemiparetic subjects: impact of Botulinum toxin injections into rectus Femoris. Neurorehabil Neural Repair.

[14] Simon SR (2004). Quantification of human motion: gait analysis—benefits and limitations to its application to clinical problems. J Biomech.

[15] Lampire N, Roche N, Carne P, Cheze L, Pradon D (2013). Effect of botulinum toxin injection on length and lengthening velocity of rectus femoris during gait in hemiparetic patients. Clin Biomech Bristol Avon..

[16] Hesse S, Krajnik J, Luecke D, Jahnke MT, Gregoric M, Mauritz KH (1996). Ankle muscle activity before and after Botulinum toxin therapy for lower limb extensor spasticity in chronic Hemiparetic patients. Stroke.

[17] Mancini F, Sandrini G, Moglia A, Nappi G, Pacchetti C (2005). A randomised, double-blind, dose-ranging study to evaluate efficacy and safety of three doses of botulinum toxin type a (Botox) for the treatment of spastic foot. Neurol Sci.

[18] Baricich A, Carda S, Bertoni M, Maderna L, Cisari C (2008). A single-blinded, randomized pilot study of botulinum toxin type a combined with non-pharmacological treatment for spastic foot. J Rehabil Med.

[19] Stroke--1989 (1989). Recommendations on stroke prevention, diagnosis, and therapy. Report of the WHO Task Force on Stroke and other Cerebrovascular Disorders. Stroke J Cereb Circ.

[20] Pradon D, Hutin E, Khadir S, Taiar R, Genet F, Roche N (2011). A pilot study to investigate the combined use of Botulinum toxin type-a and ankle foot orthosis for the treatment of spastic foot in chronic hemiplegic patients. Clin Biomech.

[21] Bensmail D, Denormandie P, Parratte B (2006). Guide des points d’injection de toxine botulinique de type A. à dire d’experts.

[22] Clarkson HM. Musculoskeletal assessment: joint motion and muscle testing. 3rd ed. Philadelphia: Wolters Kluwer/Lippincott Williams & Wilkins Health; 2013.

[23] Bohannon RW, Smith MB (1987). Interrater reliability of a modified Ashworth scale of muscle spasticity. Phys Ther.

[24] Lin FM, Sabbahi M (1999). Correlation of spasticity with hyperactive stretch reflexes and motor dysfunction in hemiplegia. Arch Phys Med Rehabil.

[25] Katz RT, Rovai GP, Brait C, Rymer WZ (1992). Objective quantification of spastic hypertonia: correlation with clinical findings. Arch Phys Med Rehabil.

[26] Shaw L, Rodgers H, Price C, van Wijck F, Shackley P, Steen N, et al. BoTULS: a multicentre randomised controlled trial to evaluate the clinical effectiveness and cost-effectiveness of treating upper limb spasticity due to stroke with botulinum toxin type a. Health Technol Assess. 2010;14 10.3310/hta14260. 10.3310/hta14260 20515600

[27] Paternostro-Sluga T, Grim-Stieger M, Posch M, Schuhfried O, Vacariu G, Mittermaier C (2008). Reliability and validity of the Medical Research Council (MRC) scale and a modified scale for testing muscle strength in patients with radial palsy. J Rehabil Med.

[28] Bohannon RW (2005). Manual muscle testing: does it meet the standards of an adequate screening test?. Clin Rehabil.

[29] Chapman AE (1985). The mechanical properties of human muscle. Exerc Sport Sci Rev.

[30] Delp SL, Loan JP, Hoy MG, Zajac FE, Topp EL, Rosen JM (1990). An interactive graphics-based model of the lower extremity to study orthopaedic surgical procedures. IEEE Trans Biomed Eng.

[31] Hatze H (1977). A complete set of control equations for the human musculo-skeletal system. J Biomech.

[32] Hoy MG, Zajac FE, Gordon ME (1990). A musculoskeletal model of the human lower extremity: the effect of muscle, tendon, and moment arm on the moment-angle relationship of musculotendon actuators at the hip, knee, and ankle. J Biomech.

[33] Pandy MG, Anderson FC (2000). Dynamic simulation of human movement using large-scale models of the body. Phonetica.

[34] Zajac FE (1989). Muscle and tendon: properties, models, scaling, and application to biomechanics and motor control. Crit Rev Biomed Eng.

[35] Arndt AN, Komi PV, Brüggemann G-P, Lukkariniemi J (1998). Individual muscle contributions to the in vivo achilles tendon force. Clin Biomech Bristol Avon.

[36] Anderson FC, Pandy MG (2003). Individual muscle contributions to support in normal walking. Gait Posture..

[37] Erdemir A, McLean S, Herzog W, van den Bogert AJ (2007). Model-based estimation of muscle forces exerted during movements. Clin Biomech.

[38] Liu MQ, Anderson FC, Schwartz MH, Delp SL (2008). Muscle contributions to support and progression over a range of walking speeds. J Biomech.

[39] Neptune RR, Zajac FE, Kautz SA (2004). Muscle force redistributes segmental power for body progression during walking. Gait Posture..

[40] Neptune RR, Sasaki K, Kautz SA (2008). The effect of walking speed on muscle function and mechanical energetics. Gait Posture..

[41] Pierce SR, Johnston TE, Shewokis PA, Lauer RT (2008). Examination of spasticity of the knee flexors and knee extensors using isokinetic dynamometry with electromyography and clinical scales in children with spinal cord injury. J Spinal Cord Med.

[42] Boudarham J, Roche N, Pradon D, Bonnyaud C, Bensmail D, Zory R (2013). Variations in kinematics during clinical gait analysis in stroke patients. PLoS One.

[43] Hermens HJ, Freriks B, Merletti R, Stegeman D, Blok J, Rau G (1999). European recommendations for surface electromyography. Roessingh Res Dev.

[44] Ivanenko YP, Poppele RE, Lacquaniti F (2004). Five basic muscle activation patterns account for muscle activity during human locomotion. J Physiol.

[45] Bizzi E, Cheung VCK, d’Avella A, Saltiel P, Tresch M (2008). Combining modules for movement. Brain Res Rev.

[46] Delis I, Panzeri S, Pozzo T, Berret B (2014). A unifying model of concurrent spatial and temporal modularity in muscle activity. J Neurophysiol.

[47] Lexell J, Flansbjer U-B, Holmbäck AM, Downham D, Patten C (2005). Reliability of gait performance tests in men and women with hemiparesis after stroke. J Rehabil Med.

[48] Eng JJ, Dawson AS, Chu KS (2004). Submaximal exercise in persons with stroke: test-retest reliability and concurrent validity with maximal oxygen consumption. Arch Phys Med Rehabil.

[49] Schmid A, Duncan PW, Studenski S, Lai SM, Richards L, Perera S (2007). Improvements in speed-based gait classifications are meaningful. Stroke.

[50] Bowden MG, Balasubramanian CK, Behrman AL, Kautz SA (2008). Validation of a speed-based classification system using quantitative measures of walking performance Poststroke. Neurorehabil Neural Repair.

[51] Alzahrani MA, Dean CM, Ada L (2009). Ability to negotiate stairs predicts free-living physical activity in community-dwelling people with stroke: an observational study. Aust J Physiother.

[52] Berg KO, Wood-Dauphinee SL, Williams JI, Maki B (1992). Measuring balance in the elderly: validation of an instrument. Can J Public Health Rev Can Sante Publique.

[53] Gallien P, Adrien S, Petrilli S, Durufle A, Robineau S, Kerdoncuff V (2005). Home care and quality of life three years after stroke. Ann Réadapt Médecine Phys.

[54] Bugge C, Hagen S, Alexander H (2001). Measuring stroke patients’ health status in the early post-stroke phase using the SF36. Int J Nurs Stud.

[55] Dodds TA, Martin DP, Stolov WC, Deyo RA (1993). A validation of the functional independence measurement and its performance among rehabilitation inpatients. Arch Phys Med Rehabil.

[56] Chumney D, Nollinger K, Shesko K, Skop K, Spencer M, Newton RA (2010). Ability of functional independence measure to accurately predict functional outcome of stroke-specific population: systematic review. J Rehabil Res Dev.

[57] Caty GD, Arnould C, Stoquart GG, Thonnard J-L, Lejeune TM (2008). ABILOCO: a Rasch-built 13-item questionnaire to assess locomotion ability in stroke patients. Arch Phys Med Rehabil.

[58] Powell LE, Myers AM (1995). The activities-specific balance confidence (ABC) scale. J Gerontol A Biol Sci Med Sci.

[59] Faul F, Erdfelder E, Lang A-G, Buchner A (2007). G* power 3: a flexible statistical power analysis program for the social, behavioral, and biomedical sciences. Behav Res Methods.

[60] Julious SA, McIntyre NE (2012). Sample sizes for trials involving multiple correlated must-win comparisons. Pharm Stat.

[61] Holm S (1979). A simple sequentially Rejective multiple test procedure. Scand J Stat.

